# Recruitment principles and strategies for supportive care research in pediatric oncology

**DOI:** 10.1186/s12874-021-01371-1

**Published:** 2021-08-28

**Authors:** Natalie Bradford, Christine Cashion, Paula Condon, Shelley Rumble, Alison Bowers

**Affiliations:** 1 Queensland University of Technology, Cancer and Palliative Care Outcomes Centre and School of Nursing, Brisbane, Australia; 2 Queensland University of Technology at Centre for Children’s Health Research, 62 Graham St, South Brisbane, Queensland 4101 Australia; 3 Oncology Services Group, Queensland Children’s Hospital, Children’s Health Queensland, Brisbane, Queensland Australia

**Keywords:** Child, Neoplasms, Research design, Research personnel, Recruitment, Barriers

## Abstract

**Background:**

Variations in clinical practice contribute to negative outcomes for children with cancer. Research in this area is imperative to standardise practice, yet such research is challenging to undertake, and a significant proportion of studies fail. A common reason for failure is poor recruitment, yet little information is available to support researchers and clinicians planning such research.

**Methods:**

Our primary aim was to describe the recruitment strategies and outcomes in a tertiary children’s hospital across multiple observational supportive care studies. Secondary aims were to establish principles to improve both recruitment strategies and the reporting of recruitment. We undertook a retrospective descriptive analysis of the recruitment logs and data from three studies in pediatric oncology. The mean time to recruit one participant was calculated. Common reasons for not approaching eligible participants and reasons potential participants declined are described.

**Results:**

Of the 235 potential candidates across all studies, 186 (79%) were approached and of these 125 (67%) provided consent, with 117 (63%) completing baseline measures. We estimated recruitment per participant required an average 98 min of experienced research nurse time. Four factors are described that influence recruitment and six principles are outlined to maximise recruitment and the generalisability of research findings.

**Conclusions:**

We highlight the recruitment experiences across three different projects in children’s cancer supportive care research and provide a roadmap for other researchers planning to undertake clinical research in pediatrics.

## Background

### Problem description

The first critical step for any clinical research is recruitment of participants. Indeed the ultimate success and translation of research findings into practice is entirely dependent on the successful recruitment of participants [1]. The highly organised structure of co-operative clinical trials in pediatric oncology are commonly supported by central administration and dedicated research staff at each institution. Significant efforts are made to enrol as many children as possible to receive anticancer treatment through a clinical trial [2]. In comparison, there are numerous barriers recruiting to supportive care research including: difficulties obtaining ethical approvals, logistical challenges with identification and contact with eligible participants, clinician gatekeeping, perception of potential burden and less available funding and resources compared to cancer treatment clinical trials [2, 3]. As a consequence, many supportive care studies fail to meet their anticipated recruitment rate.

### Available knowledge

Supportive care is a broad term defined by the Multinational Association of Supportive Care in Cancer as “the prevention and management of the adverse effects of cancer and its treatment. This includes management of physical and psychological side effects and symptoms across the continuum of the cancer experience from diagnosis through treatment to post-treatment care.” [4] The terms supportive care and palliative care are often used interchangeably as both are dedicated to enhancing quality of life by preventing and managing symptoms, and are underpinned by good communication between children, families and healthcare providers. The term supportive care is used throughout this paper and refers to care provided to manage the short- and long-term physical and psychological effects of cancer treatment. Such care is critical to minimise sequelae of treatment related late effects, and should be evidence-based to achieve standardisation [5]. Progress to achieve standardisation in supportive care however, is not well developed compared with anticancer treatment research [5]; there are variations in clinical practice both within and between different institutions and across different nations [3, 6–9]. As a result, an estimated 30–50% of children with cancer do not receive optimal care with variations in practice contributing to negative outcomes [10, 11]. These include treatment-related and growth and development arrest resulting in significant morbidity and mortality [12, 13]. With cancer diagnostics and treatment constantly evolving, it is imperative supportive care research keeps up to address the adverse effects of cancer treatment and improve outcomes and experiences of patients, families, and health services [11].

### Rationale

Understanding successful recruitment strategies is therefore an imperative for advancing supportive care research. A recent systematic review of 215 studies involving children with life threatening illness highlighted all stages of recruitment were consistently underreported. This limits the knowledge of successful recruitment strategies, reduces quality, impedes ability to judge the applicability of findings, and contributes to research that does not add to existing knowledge- known as research waste [14, 15].

To address this issue, practical strategies to improve recruitment needs to be established and shared, and reporting of all levels of recruitment should be transparent [16]. Considering the slow progress of supportive care research in children with life threatening illnesses such as cancer, evidence is required to support research teams to maximise recruitment and thereby advance this field. Our primary aim therefore was to describe the recruitment strategies and outcomes in a tertiary children’s hospital across multiple observational supportive care studies. Secondary aims were to establish principles to improve both recruitment strategies and the reporting of recruitment.

## Methods

### Context

We undertook a retrospective analysis of the recruitment logs and data from three observational studies in pediatric oncology. These three studies were selected for pragmatic reasons; all three were undertaken within a few months of each other by the study authors, and they provided diverse recruitment experiences. NB is a clinician researcher, and previous oncology nurse who designed and led all studies. CC and PC are research nurses responsible for recruitment with extensive clinical oncology experience and were not involved in the clinical care of study participants. SR is a clinical nurse who is involved in clinical care but did not participate in recruitment or data collection. AB is a clinician researcher with expertise in research ethics and governance. All studies were undertaken at a single site, a large tertiary children’s hospital in a large metropolitan city in Australia. The cancer service receives approximately 220 new cancer diagnoses in children and adolescents (0–18 years) each year and is an accredited Children’s Oncology Group member facility. All three studies used validated surveys for measures where possible. Across all three studies a recruitment period of approximately 6-12 months was evaluated. Research nurses responsible for recruitment were available approximately 2 days per week for each study. During periods of lockdown because of the COVID-19 pandemic, recruitment was paused for studies 1 and 2 (study 3 had completed planned recruitment).

### Ethics and consent

All studies were approved by the Children’s Health Queensland Human Research Ethics Committee (HREC/18/QRCH/18; HREC/19/QCHQ/53816; LNR/18/QCHQ/48237). No additional amendments were required for this paper. Written informed consent was obtained from the parents or guardians of the children who served as subjects of the investigation and, when appropriate, assent from the children themselves. All study processes and methods were carried out in accordance with relevant guidelines and regulations.

### Overview of studies

#### Study 1: Remote Symptom Management in Pediatric Oncology (RESPONSE)

The RESPONSE study included children (3–18 years) with any diagnosis of cancer except brain cancer, receiving a cycle of chemotherapy and their family caregiver. This phase of RESPONSE was an exploratory observational study investigating cancer treatment related symptoms and the feasibility of routine use of Patient Reported Outcome Measures (PROMs). Children (or parent proxy) completed weekly surveys to measure total symptom burden (SSPedi) [17, 18], symptom intensity and distress (FACES pain scale) [19] and quality of life (PedsQL-Cancer) [20] over eight consecutive weeks. Recruitment logs from 30 Jan 2020 through 29 Jan 2021 were included in this analysis.

#### Study 2: Child and Adolescent Family Experiences of Brain Cancer (CASPER)

The second study included family caregivers of children (0–18 years) with brain cancer. Family caregivers were recruited approximately 6–12 weeks following their child’s diagnosis to participate in a longitudinal observational study, completing a series of questionnaires over a two-year time period to measure: child and family caregiver quality of life (PedsQL-Brain Cancer [21] CQoLC [22]), impact of illness on family functioning (McMaster Family Functioning Device [23]), financial toxicity (survey developed for study) and care integration (Pediatric Integrated Care Survey [24]). Recruitment logs from 25 Feb 2020 through 24 Feb 2021 were included in this analysis.

#### Study 3: Assessing Modifiable Health Behaviours after Cancer (AMBER)

The third study recruited survivors (> 5 years disease free) of any childhood cancer attending long term late-effects follow up to an observational cross sectional study. Survivors completed an in-depth survey about their symptoms (PRO-CTCAE [25]), health behaviours (self-report survey developed for study), quality of life (FACT-G [26]) and self-efficacy managing their health (PROMIS Self- Efficacy [27]). They wore an activity tracker for 2 weeks and completed diaries about their food and beverage intake. Recruitment logs from 20 March 19 through 18 September 2019 were included in this analysis.

### Analysis

The screening and recruitment logs for each study, documented in excel spreadsheets, were descriptively analysed. Data are presented regarding the numbers of potential participants screened, approached, missed, consented, and those ultimately participating. Data are presented with means and ranges where possible. Common reasons for not approaching eligible participants and reasons potential participants declined are described. Additional data were collected for study 1 tabulating the pre-screening process over a four-week period to summarise: the number of patients screened per day; those deemed eligible and ineligible; the time taken to screen (both through electronic medical records and with clinical staff), and the number of participants recruited. The mean time to recruit one participant was estimated by dividing the total time taken to pre-screen and approach potential participants by the number successfully recruited.

## Results

Of the 235 potential candidates across all three studies, 186 (79%) were approached and of these 125 (67%) consented, with 117 (63%) completing baseline measures (Table 1). All participants were recruited from the hospital, either from outpatient clinics or via telephone from eligible patient lists provided by clinical nurse consultants. Participants were recruited by experienced oncology research nurses, who understood patient’s clinical status and who were able review medical records and liaise with clinical staff as required. The research nurses pre-screened all participants to ensure those who were ineligible or where it was deemed inappropriate to approach, were not approached. This reduced potential burden on families and clinical staff.

**Table 1:** Overview of recruitment to included studies

	Study 1 RESPONSE		Study 2 CASPER		Study 3 AMBER	
	Child 3–18 years, any cancer except brain, at start of chemotherapy cycle		Child 0–18 years newly diagnosed (within last 6–12 weeks) with brain cancer		Survivors of any type of childhood cancer > 5 years post treatment	
	N	%	N	%	N	%
**Eligibility criteria**						
Identified as eligible	96	100%	82	100%	57	100%
Approached (% of eligible)	75	78%	54	66%	57	100%
Consented (% of approached)	49	65%	45	83%	31	54%
Completed baseline (% consent)	46	94%	41	91%	30	97%
**Reasons eligible candidates not recruited**						
Clinician request not to approach (% eligible)	18	19%	15	18%	0	0%
Missed in clinic/ unable to contact (% eligible)	4	4%	0	0%	6	11%
Approached and declined (% eligible)	26	27%	2	2%	9	16%
Consent given but did not complete baseline (% consent)	3	6%	4	5%	1	2%

### Pre-screening

Pre-screening refers to evaluation of the eligibility of the potential participant to determine suitability and the appropriate timing to approach. In all three studies research nurses employed by the hospital facility, but renumerated through research funds, used electronic hospital administration systems to review the patients attending clinics, and then to screen for eligibility against patient individual medical records. All potential participants were then briefly discussed with clinical nursing and/or medical staff to confirm it was appropriate to approach each patient/family; this process was facilitated by the research nurse attending schedule meetings. An overview and example of pre-screening processes and estimates of time and other outcomes is presented in Table 2.

**Table 2:** Pre-screening process outcomes over 4 weeks from study 1 RESPONSE

Pre-screening process outcomes	
Number of active days of recruitment	Total = 8 days
Number patients screened per day	Mean = 25, range 19–37
Number eligible patients identified per day	Mean = 6, range 3–9
Number ineligible patient identified per day	Mean = 20, range 13–30
Time taken to screen patients per day	Mean = 56 min, range 36–79
Time taken consulting with clinician nursing staff per day	Mean = 7 min
Time to approach potential participants	Mean = 30 min, range 10–40
Number of patients recruited	Total = 7 patients
Estimated time taken to recruit one patient	Mean = 98 min

As the research nurses across all studies worked in a part-time capacity in research roles, there were days when potentially eligible participants were not approached. This contributed to varying numbers of potential participants being approached (66–100%, Table 1) depending upon each studies’ eligibility and what clinics participants were recruited from. For example, study 3 recruited survivors from one clinic, that ran each Wednesday, and the research nurse was available to approach every participant identified as eligible. Whereas in study 1, participants were recruited from the general oncology outpatients’ clinics, which ran 5 days per week, however the research nurse worked only 2 days per week. By keeping accurate logs of activity, most eligible participants (67%) were still able to be recruited. The process of trying to ‘catch’ potential participants in between clinical encounters is, however, time consuming and requires intimate knowledge of the structures and processes undertaken in oncology clinics, as well as an understanding of individual family dynamics. Research nurses had previously worked in the oncology outpatients clinic and were familiar other medical, nursing and administrative staff. Having research nurses familiar with the department likely reduced clinician gatekeeping and research nurses were able to meet with patients and families before or after clinic appointments. When this was not possible, patients and families were followed up with email, text messages or phone calls. Participants were given time to read information sheets, discuss participation with significant others, ask questions and consider participation. Choice was given regarding the method of participation including electronic surveys via REDCap [28] (available on mobile phones, tablets and computers), paper surveys, or completion with the research nurse over the telephone. This flexible approach however, meant there could be delays with recruiting participants and extra research nurse time required to follow up potential participants at subsequent clinic visits. For some candidates, despite apparent willingness to participate, and multiple contacts, they ultimately chose not to participate. The below notes from recruitment logs exemplify this:

Visited OPD for introduction. Appointment had been delayed and rescheduled, missed family. Visited one week later at rescheduled appointment and introduced parent to research and left paper information. At parent request, visited next day in ward, discussed further, answering questions. Parent consented and sent email link same day. No response 1 week after consent. Followed up with phone call, parent had not seen email but indicated had a lot going on at the moment. Given the option to withdraw but parent said they would check later in the day. No response two weeks after consent, not pursued any further [Study 1]

### Reasons deemed inappropriate to approach

Clinical nursing, medical staff, or the research nurses themselves, made decisions it was not appropriate to approach children and parents to participate in research for several reasons (Table 1). The most common reason was due to unstable disease or disease progression. Another common reason was because of complex family dynamics, and highly anxious families. Families who did not speak, read and understand fluent English were also reasons families were not approached.

### Reasons for declining to participate

Families volunteered reasons for declining participation. The most common reason (46%) was because a parent felt there was too much going on - either because of the complexities of cancer treatment, or family life. Some families reported feeling overwhelmed and not having capacity to consider taking on the extra burden of participation in research (Table 3). A few parents and children reported the study aims were not relevant to them - this was particularly the case for children closer to the end of treatment who were feeling less bothered by symptoms and treatment and who were looking forward to the end of planned treatment.

**Table 3:** Reasons for declining participation

Reasons for declining participation RESPONSE study	N	%
Too much going on	12	46%
Study not relevant /not interested	5	19%
No reason given	4	15%
Difficulties with shared custody and timing of study measures	2	8%
Non-verbal child- questions difficult to provide proxy answer	2	8%
Already participated in several projects, does not want to participate in any more	1	4%
*Total*	*26*	*100%*

## Discussion

There are numerous challenges recruiting children, young people and their parents to research studies in the face of serious life-threatening illness and few examples of successful recruitment strategies, or indeed recruitment rates in supportive care pediatric oncology research [16, 29]. Yet the success of any research is dependent upon the ability to recruit the required number of participants within the timeframes of the study. We report here our experiences with recruitment across three observational supportive care studies in pediatric oncology. We identified varying recruitment rates between 54 and 83%, which is higher than the range (47–65%) of studies that report recruitment rates in adolescent and young adult supportive care studies [1]. Taking into account time for pre-screening and approaching participants, we estimate it takes an average of 98 min for an experienced research nurse to screen, approach and recruit one participant. It is common to underestimate the time required, human resources needed and costs associated with recruitment [30] and these data provide pivotal information for other researchers embarking on research in this population.

Previous research describes four factors considered influential in recruitment of participants: 1) infrastructure, 2) the nature of the research, 3) recruiter characteristics and 4) participant characteristics [31]. Processes to address each factor are described in turn below.

Factors related to infrastructure include developing systems and processes to manage data, including a pre-screening protocol and tracking procedure. These assist with identifying potential participants; establishing eligibility; identifying the optimal time to approach; tracking consent and enrolment, refusal, and attrition. In addition, a communication strategy including regular meetings, correspondence and shared files can assist with communication between investigators and research staff. All research processes, including recruitment outcomes should be reported with transparency and consistency using reporting guidelines, so that readers can draw their own conclusions about the generalisability and applicability of the research to their own setting [14].

To overcome factors related to the second factor – the nature of the research*,* it is increasingly recommended to co-design supportive care research with patients, families and clinicians [32]. Co-design increases the visibility of research, and ensures the research is relevant, acceptable and appropriate. Moreover, co-design promotes collaborative relationships with clinicians, and facilitates open communication throughout the recruitment process that can reduce gate-keeping [33]. In recognition of the importance of including consumers perspectives, funding bodies are also commonly including criteria in grant applications regarding patient and public involvement [34].

We propose the third factor, recruiter characteristics, are not as important as recruiter *processes*. Previous research has reported participants are more likely to consent if they are asked first by medical staff, even if a research nurse provided more detailed information and obtained consent [31]. Care needs to be taken to ensure patients do not feel undue pressure to participate, which may be related to unequal and dependant relationships [35]. We argue it is important for recruiters, regardless of discipline, to receive adequate training and support and to establish sound processes. Training should include discussion of the purpose of the study, description of roles and responsibilities and expectations, and team building activities to encourage the sense of belonging to a team doing improvement work [1]. Processes should outline who, how and when potential participants are approached. In the studies described here, pre-screening occurs to first identify eligible participants attending clinic. First preference is for the research nurse to recruit from the outpatient oncology clinic face-to-face. A large iPad is available for participants to complete survey measures at the time of consent through REDCap [28]; this can reduce burden. Where this is not possible, telephone and/or text messaging is used to contact families. If email addresses have been provided (and consent to contact via email is provided) these are used to send electronic links to complete surveys. Additionally, choice is provided regarding the method of participation, as well as electronic surveys, paper copies of surveys are available, or the research nurse can take answers over the telephone to complete the survey on the participant’s behalf.

In our experience, we identified a stepped rather than discrete process for recruitment as the most effective approach for recruiting participants from hospital settings [36]. In this approach, verbal and non-verbal cues are observed, and information provided accordingly. For instance, if the research nurse observes the parent is stressed, they may be provided with written information only and the research nurse will approach again later. Some participants request time to discuss with partners and other family members, and if this is the case, the research nurse will follow up with a phone call, or again later in clinic. While these processes are more time consuming, with participants contacted multiple times, our findings support this use of such processes. We established recruitment per participant required an average 98 min. Considering the efforts in developing research protocols and interventions, obtaining regulatory approvals, and managing data, as well as undertaking analysis and dissemination, the time taken to recruit participants is a critical investment and should be considered when developing budgets.

The final factor considered to influence recruitment is the participant characteristics [31]. We identified the most common reason parents decline participation is because of the intensity of treatment and caring for a sick child, with parents reporting feeling overwhelmed. Commonly, in the RESPONSE study this was when the child was experiencing distressing symptoms. The stepped recruitment process described above can address some of these barriers by providing multiple opportunities for recruiters and participants to discuss the proposed study. Conversely, a number of parents declined participation because their children were closer to the end of their treatment, experiencing less symptom burden and they felt more in control. Anecdotally, our RESPONSE study explores feasibility of symptom management using a PROM, and the paradox is families who participated in the study were often further along the treatment trajectory and frequently stated the systematic process of recording and monitoring symptoms using the PROM would have been useful *‘in the beginning when things were bad’.* However, families early in treatment felt overwhelmed by the newness of cancer and treatment and were less likely to participate. The research nurse can play a crucial role here, providing information, including other participants feedback, which may help families understand what participation in research may involve. Future planned research will explore this phenomenon further and work with families to develop greater understanding of the appropriate timing for approaching families and to develop resources to support informed decision making.

**Fig. 1:**
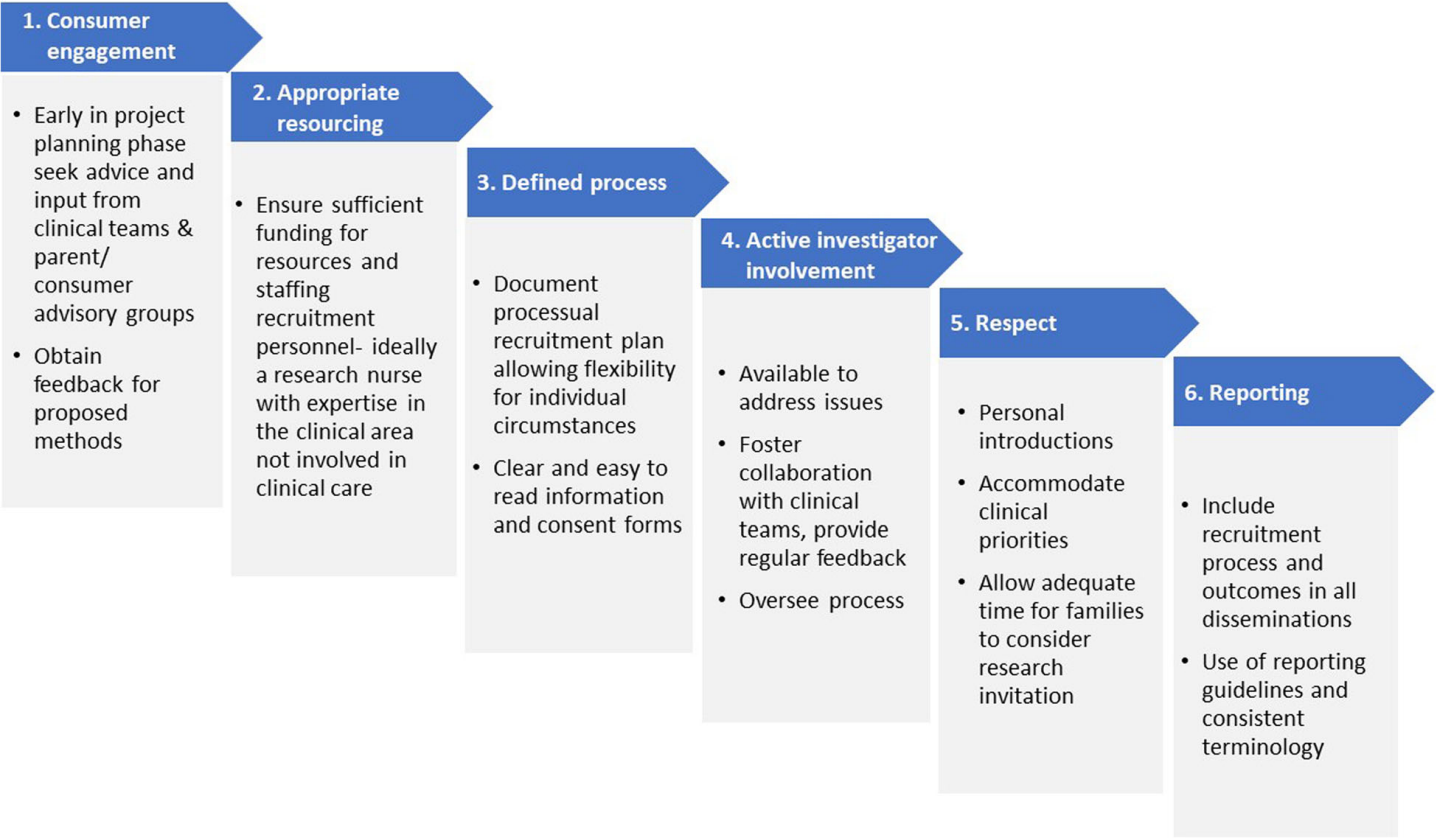
Principles and strategies to maximise the success of research to contribute towards evidence

International research across other pediatric specialities identified as much as 40% of studies are discontinued with the main reason cited as due to slow recruitment [37]. Multivariate analysis identified pediatrics was not an independent factor; non-industry funding, the acute care setting and smaller sample sizes elevated the risk for discontinuation [37]. Building upon recruitment strategies described in other fields [38], we recommend following the principles outlined in Fig. 1 to maximise the success of research to contribute meaningful and generalisable evidence. These steps encompass the four factors described earlier related to successful recruitment, and extend to summarise six broad principles to maximising recruitment:

1. Consumer engagement to ensure the research is relevant, appropriate and acceptable
2. Appropriate resourcing – ensuring adequate funding has been allocated for recruitment
3. Defined processes- documented procedures allowing for flexibility
4. Active involvement of research investigators
5. Respect for both families and clinicians
6. Reporting of recruitment rates and processes in dissemination

### Strengths and limitations

This paper adds to the scant literature available regarding recruitment of children and families with life threating illnesses such as cancer to observational supportive care research. We have described processes for recruitment and provide guidance for other researchers to anticipate challenges and minimise research waste. We acknowledge limitations, including that the data used to calculate the time taken to recruit patients is based on recruitment logs that were not developed for this purpose. Moreover, we present experiences from a single institution and from only three studies with small sample sizes; this limits the generalisability of our estimations. Recruitment to randomised clinical trials in supportive care would likely have additional barriers such as extra time required for random treatment allocation. Future research is planned to work with families to explore and refine the principles and strategies outlined here, and to further develop best-practice recruitment strategies.

## Conclusion

As the recognition of the importance of supportive care research grows, the culture of research is shifting to recommend an integrated approach that balances research focussed on the pinnacle of cure with the broader needs of patients. We have outlined the principles to optimise recruitment to observational supportive care research. This information may be useful beyond pediatric oncology as the principles outlined here are relevant across all specialities and age groups. Following these principles will help ensure results obtained are valid, generalizable and that outcomes are relevant to individuals, clinicians, and health services.

## Data Availability

Data sharing not applicable to this article as no datasets were generated or analysed during the current study.

## Abbreviations

PROM: Patient Reported Outcome Measure
SSPedi: Symptom Screening in Pediatrics
HREC: Human Research ethics Committee
LNR: Low Negligible Risk
PRO-CTCAE: Patient Reported Outcome Common Terminology Criteria for Adverse Events
FACT-G: Functional Assessment of Cancer Therapy General
PedsQL Cancer: Pediatric Quality of Life Cancer
